# Lower Health Literacy of Mania Than Depression Among Older People: A Random Survey of a Community Healthcare Service Center

**DOI:** 10.3389/fpsyt.2021.512689

**Published:** 2021-03-11

**Authors:** Leping Huang, Ruyan Huang, Yue Fei, Taosheng Liu, David Mellor, Weiyun Xu, Jinxia Xiong, Rongjie Mao, Jun Chen, Yiru Fang, Zhiguo Wu, Zuowei Wang

**Affiliations:** ^1^Division of Mood Disorders, Hongkou District Mental Health Center, Shanghai, China; ^2^Department of Psychology, Naval Medical University, Shanghai, China; ^3^School of Psychology, Deakin University, Burwood, VIC, Australia; ^4^Division of Mood Disorders, Shanghai Mental Health Center, Shanghai Jiao Tong University School of Medicine, Shanghai, China; ^5^Department of Psychiatry and Neuropsychology, Shanghai Deji Hospital, Qingdao University, Shanghai, China; ^6^School of Medicine, Shanghai University, Shanghai, China

**Keywords:** health literacy, depression, mania, older people, community healthcare service

## Abstract

**Purpose:** This study examines health literacy among older outpatients in two Community Healthcare Service Centers in Shanghai, China to facilitate the design of public education programs for the aged population on mood disorders (both depression and mania).

**Patients and Methods:** A total of 173 outpatients aged 60 years or more with a chronic physical illness were randomly sampled. A health literacy questionnaire was used to assess participants' awareness of depression and mania. Participants were then asked to label two vignettes depicting depression and mania and to give their recommendations for how to seek help for those in the vignettes and how mood disorders should be managed.

**Results:** In all, 86.1 and 36.4% of participants had heard of depression and mania, respectively, with the most common source of information being relatives and friends. Over half of the participants attributed the possible causes of mood disorders to psychological trauma, pressure or stress in daily life, taking things too hard, and personality problems. Almost two-thirds of participants correctly labeled the depression vignette, but only 26.6% correctly labeled the mania vignette. The most common methods recommended by the participants as being helpful for the individuals portrayed in the vignettes were “traveling” and help-seeking from a psychological therapist/counselor, a psychiatrist, or a close family member or friend.

**Conclusion:** The older individuals attending community healthcare service settings in Shanghai have good depression literacy but relatively poor mania literacy. However, most participants had a positive attitude toward psychiatric treatment for mood disorders.

## Introduction

The rapid increase in the world's aging population has led to a growing body of research focused on the mental health of older people. A major focus of this research has been mood disorders, with studies reporting prevalence rates between 10 and 38% for unipolar depression and 0.1 and 0.5% for bipolar disorders (1). In China, the prevalence of mood disorders lasting longer than 1 month among the older community population (10.56%, aged ≥55 years) has been reported to be higher than for the younger population (7.72% for those aged 40–54 years and 3.85% for those aged 18–39 years) (2). In contrast, the prevalence of bipolar disorder in the older populations (0.4% for those aged 50–64 years and 0.1% for those aged ≥65 years) in China is moderately lower than that of younger populations (0.6% for those aged 35–49 years and 0.5% for those aged 18–34 years) (3).

For elderly (aged ≥60 years) patients with different medical illnesses, the prevalence of depression increases dramatically (4). We have previously reported that 35.2% of elderly outpatients (aged 63.2 ± 11.8 yeas) with a medical illness attending a Community Healthcare Service Center in Shanghai had depressive symptoms, and 16.1% of the participants met the criteria for major depressive disorder (5). Of the comorbid conditions, those with Parkinson's disease appeared to exhibit the highest prevalence of depression (up to 75%) (6). Geriatric patients with bipolar disorders are likely to have more than one comorbid medical condition (7), although less is known about the prevalence of bipolar disorders among elderly people with medical illness (4).

Accurate diagnosis and appropriate treatment are critical for the optimization of both clinical efficacy and functional outcomes for older people with depression or bipolar disorder (8–10). However, mood disorders can be particularly difficult to diagnose in elderly patients; this is due not only to their multiple somatic and cognitive symptoms but also to negative attitudes toward illness (e.g., stigma), which have been found to be major obstacles to help-seeking among patients with mood disorders and their families in either Western (11–13) or in Chinese settings (2, 14–16). Furthermore, it has been reported that both older members of the general population (13, 16) and elderly patients with medical illness (17) have lower depression literacy than the younger population and are more likely to prefer informal treatments such as family support (13, 18) and herbal medicine (18). This suggests this vulnerable population might be less likely to be aware that their mood problems are in fact symptoms of an illness that can be treated. Even if they did understand this, they are likely to have a negative attitude toward formal psychiatric diagnosis and treatment.

To date, there has been no specific survey on both depression and mania literacy among elderly people in China. In particular, little is known about their awareness of mania or bipolar disorders, especially the more vulnerable population with medical conditions. More research is needed in this area given the very low levels of help-seeking from patients with mood disorders (2) and frequent misdiagnosis and underdiagnosis of bipolar disorders (19).

The current study therefore aims to assess the health literacy of mood disorders (including depression and mania) among older outpatients with medical conditions in the Community Healthcare Service Centers of Shanghai. It is expected that the level of knowledge and beliefs about bipolar disorders may differ from those of depression.

## Materials and Methods

### Subjects

The survey on health literacy of depression and mania was conducted in the Hongkou District of Shanghai. The target sample size was determined by assuming that the rate of correct identification for depression in Shanghai would be 12% (15) with a setting α = 0.05 and estimation accuracy error σ = 5%, which would require the sample size to be 162 using the formula for the estimation of population rate. Based on the assumption of a 90% response rate, this study would require us to survey 178 individuals. At first, we randomly selected two of eight Community Healthcare Service Centers in Hongkou District and then sampled one out of every 30 outpatients with chronic physical diseases in the two centers selected. If they were interested in the survey, the sampled participants were introduced briefly to the study's purpose and procedures and informed they could withdraw at any time during the data-collection process should they wish to do so.

The study was approved by the Hongkou Mental Health Center Medicine Ethics Committee, Shanghai, China. Written informed consent was obtained from each participant before any study-related procedure was implemented. If the eligible participants had any difficulty in understanding and signing the informed consent, written informed consent was also obtained from their guardians. All participants were given a set of kitchen utensils (valued at about USD $5) as reimbursement for their participation.

Data were collected between June 9 and July 28, 2018. Inclusion criteria included being at least 60 years of age (15) and being a registered resident of Shanghai. Exclusion criteria included a diagnosis of dementia or severe cognitive dysfunction. All participants participated in face-to-face interviews. Socio-demographic and clinical data were recorded by research psychiatrists. A self-rating questionnaire was used to evaluate participants' literacy for mood disorders. All the participants completed the questionnaire by themselves. If the participants had difficulty in understanding the questions or answer options, the research psychiatrists, who were specifically trained for this project, were allowed to give flexible interpretation by using Mandarin Chinese or regional dialects but were not allowed to help the participants to make any choice.

### Mood Disorder Literacy Questionnaire

A health literacy questionnaire (Chinese version) consisting of a series of fixed choice questions was used to assess participants' recognition of mood disorders. It was based on the concept of Mental Health Literacy (20) and adapted from the Mental Health Literacy Scale (21) by the research team. The content included in the questionnaire has been validated in several studies in China and in our previous studies (15–18). Aspects of health literacy assessed included awareness of mood disorders, ability to label vignettes depicting depression and mania, beliefs about helpfulness of different professionals and coping activities, and preference for psychiatric treatment for dealing with mood disorders.

The questionnaire was composed of two sections. In the first section, the following aspects of participants' awareness of mood disorders were assessed. First, we looked at the participants' understanding of the meaning of mood fluctuation (core features of mood disorders) (Item: *Is it possible that persistent mood fluctuation/instability lasting 1 or 2 weeks or even longer may indicate psychological problems or mental disorders?*). Second, we looked at the participants' awareness of named disorders. In this part, the participants were asked if they have ever heard of the terms (*mood disorders, depression, mania, hypomania*, and *bipolar disorder*). For the sake of addressing cross-linguistic differences in the understanding of the key terms, some terms were labeled by using both formal medical terms and local lexicons usually used as everyday speech by people. For example, we used “抑郁症” *(“yi yu zheng*” *in Chinese used as a formal medical term)* and “忧郁症” *(“you yu zheng*” *in Chinese usually used as everyday speech by people)* to label the term of depression or a depressive episode and “躁狂症” *(“zao kuang zheng*,” *the formal medical term) and* “狂躁症” *(“kuang zao zheng,” everyday speech)* to label the term of mania or a manic episode. Third, we looked at the participants' access to information about mood disorders (Item: *Where have you ever got access to the information about mood disorders, depression, mania, hypomania, or bipolar disorder?*). Last, we looked at the participants' understanding of possible causes of mood disorders (Item: *What do you think about the possible cause of mood disorders?*) (see Table 1 for details about the response format of the items).

**Table 1:** Percentage of participants' awareness of mood disorder (*N* = 173).

**Question**	***n***	**%**
Is it possible that persistent mood fluctuation/instability lasting for 1 or 2 weeks or longer may indicate psychological problems or mental illness?		
Probably	38	22.0
Possibly	74	42.8
Uncertainly	13	7.5
I don't know	48	27.7
Have you ever heard of the following terms? (Multiple-choice question)		
Mood disorders	36	20.8
Depression	149	86.1
Mania	63	36.4
Hypomania	15	8.7
Bipolar disorders	18	10.4
I don't know the above terms	10	5.8
Where have you ever got access to information about mood disorders, depression, mania, hypomania, or bipolar disorder? (Multiple-choice question)		
Relatives and friends	79	45.7
Television and radio broadcasting	56	32.4
Paper-based media	36	20.8
Community health education	30	17.3
Internet	16	9.2
What do you think are the possible causes of mood disorders? (Multiple-choice question)		
Psychological trauma	158	91.3
Pressure or stress in daily life	124	71.7
Taking things too hard	116	67.1
Personality problems	103	59.5
Fatigue	70	40.5
Heredity	63	36.4
Brain disease	53	30.6
Bewitched or demon possession	27	15.6
I don't know	19	11.0

In the second section, the participants' abilities to recognize depression and mania were evaluated with the two vignettes developed by two psychiatrists based on clinical experience and published vignette studies (15, 16, 22). The two vignettes were written to satisfy the ICD-10 diagnostic criteria for a depressive episode and a manic episode. The depressive episode vignette was as follows:

*During the last weeks, Linlin complains the following discomforts almost every day: depressed, cyring a lot, heavily tired, decreasing in activities and verbal, loss of interest or pleasure in almost everything, low appetite and decrease in weight, insomnia, worthlessness and recurrent thoughts of death and suicidal ideation or even desire of killing herself*.

The mania episode was as follows:

During the last weeks, Dawei presents with persistently elevated mood, increased activity and energy, and decreased need for sleep and feeling fully rested after only 2 or 3 h of sleep. He is more talkative than usual and boasts of his non-existing invention of a new fuel and claims that he is becoming a billionaire and has won the Nobel Prize. Which of the following diseases might best explain his situation?

After reading the vignettes, the participants were asked to make selections from the available options (for details, see **Tables 3**, **4**) to assess their beliefs about the helpfulness of different professionals and coping activities and their preference for psychiatric treatment, including psychotropic medication treatment, psychotherapy, and a combination of both, for dealing with mood disorders.

### Statistical Analysis

Statistical data management and analyses of the data were carried out using Statistical Package for Social Sciences (SPSS) Version 20.0 for Windows. The sociodemographic characteristics, depression and mania literacy, awareness of mood-disorder-related knowledge, and beliefs about professional help and treatment methods were summarized with descriptive statistics. The differences in the percentage of participants giving correct labels to the vignettes of depression and mania stratified by gender (male and female) was analyzed usinga Pearson Chi-square Test. Statistical significance was set to *p* < 0.05.

## Results

### Characteristics of Enrolled Participants

A total of 180 participants were enrolled, and 173 (96.1%) of them completed the questionnaires. Of the participants, 68.8% were female (*n* = 119). The range of age and the mean age of the participants was 60–90 years and 70.37 (*SD* = 7.76) years.

### Awareness of Mood Disorders

As shown in Table 1, 38 (22.0%) and 74 (42.8%) participants thought that persistent mood fluctuation probably indicated psychological problems or mental illness, respectively. However, over one-third of them answered that they were *uncertain* or did not know. Most of the participants (86.1%) had heard the term *depression*, but only 20.8 and 36.4% of them had ever heard the terms *mood disorders* and *mania*. The majority of them were unaware of the terms *hypomania* or *bipolar disorders*. More than half of the participants accessed information about mood disorders through traditional media (i.e., 32.4 and 20.8% for television/radio broadcasting and paper-based media, respectively.). Nearly half (45.7%) of them sourced information through their relatives and friends. Community health education and internet resources had less of an impact on the acquisition of such information. When it comes to their understanding of the possible causes of mood disorders, the majority of the respondents chose multiple causes: psychological trauma (91.3%), pressure or stress in daily life (71.7%), taking things too hard (67.1%), and personality problems (59.5%). About one-third of the participants considered that medical factors such as heredity issues (36.4%) or brain disease (30.6%) might play a role in the illness.

### Percentage of Participants Giving Correct Labels to the Vignettes

Over half of the participants (65.9%) correctly labeled the vignette depicting major depression (see Table 2). By contrast, the proportion of participants correctly labeling the mania vignette (26.6%) was much lower, with nearly 40% mislabeling it as schizophrenia and 16.8% labeling it unknown. Overall, only 21.4% (*n* = 37) of the participants correctly identified both of the vignettes. There were no significant differences for the correct rate of labeling the depression and mania vignettes between genders (all *p* > 0.05, data not shown).

**Table 2:** Percentage of participants giving labels to the depression and mania vignettes.

**Vignettes**	**Label**	***n***	**%**
Depression vignette	Normal	2	1.2
	Physical problems	0	0
	Depression	114	65.9
	Anxiety	22	12.7
	Neurasthenia	13	7.5
	Dysthymia	5	2.9
	I don't know	17	9.8
Mania vignette	Normal	4	2.3
	Physical problems	1	0.6
	Mania	46	26.6
	Schizophrenia	69	39.9
	Anxiety	15	8.7
	Neurasthenia	9	5.2
	I don't know	29	16.8

### Participants' Beliefs About Helpfulness of Different Professionals and Coping Activities and Their Preference for Psychiatric Treatment for Dealing With Mood Disorders

Similar proportions (41.0–42.2%) of the participants endorsed engagement of a psychiatrist, close family/friend, and/or a psychological therapist/counselor as helpful for the persons depicted in the vignettes. Nearly half (47.4%) of the participants endorsed the use of “traveling” as a helpful coping activity. A small proportion of the participants endorsed non-psychiatric professionals such as traditional Chinese medical doctors, physicians, and neurologists (5.8–26.0%) (*n* = 10, 5.8%) as helpful (see Table 3).

**Table 3:** Percentage of participants rating each professional and coping activity as “helpful” for the person described in both two vignettes.

	***n***	**%**
Professions		
Psychological therapist/counselor	73	42.2
Psychiatrist	71	41.0
Neurologist	45	26.0
Traditional Chinese medical doctor	15	8.7
Physician	10	5.8
Close family and friend	71	41.0
Coping activity		
Traveling	82	47.4
Getting more rest	24	13.9

If the individuals depicted in the vignettes were diagnosed with mood disorders (depression or mania), the majority of the participants (71.1%) thought that pharmaceutical approaches, in particular the “long-term psychotropic medication treatment” and “combination therapy of medication treatment and psychotherapy,” would be the most useful treatment (see Table 4). A lower percentage of the participants (26.0%) preferred “psychotherapy or psychological counseling.” Very few participants considered psychiatric treatment to be useless (2.9%).

**Table 4:** Percentage of participants' preference to psychiatric treatment for dealing with mood disorders.

	***n***	**%**
Medication treatment	123	71.1
Long-term psychotropic medication treatment	113	65.3
Short-term psychotropic medication treatment	10	5.8
Psychotherapy or Psychological counseling	45	26.0
Combination therapy of medication treatment and psychotherapy	112	64.7
Psychiatric treatment useless	5	2.9

## Discussion

This is the first study to our knowledge to investigate the health literacy of mood disorders (including both depression and mania) among older outpatients with chronic medical conditions attending community healthcare service centers in China. The findings showed that more than half of the participants did not show good comprehension of the clinical picture and etiology of mood disorders, and the participants had lower health literacy for mania compared with depression. Thus, they did not choose appropriate treatments for the person depicted in the vignettes.

Because it has a higher prevalence rate and has been discussed more frequently by the media than other disorders, depression has been shown to be one of the mental disorders with the highest rates of correct identification (23–25). In our study, almost two-thirds (65.9%) of participants correctly labeled the depression vignette, which is similar to the recognition rate of depression in Hong Kong (65.7%) (24) but lower than that found in an Australian national survey of mental health literacy (75%) (25). In contrast, a previous comparative study found that there was a much lower percentage of correctly labeling a depression vignette by Chinese people living in Shanghai (12.2%) compared to those living in Hong Kong (13.9%) and Melbourne (14.0%) (15). Two other surveys in Beijing and Liuyang city in Hunan Province found the correct labeling rate of a depression vignette to be 25.5 and 16.1%, respectively (16, 23). The differences between these studies' findings and ours could be attributed to the study population. The studies by Yu et al. (16) recruited participants from rural areas, where there are far fewer mental health resources and information compared to metropolitan cities like Shanghai. Another reason for the differences could be that the other two studies were conducted before the *2015–2020 National Mental Health Work Plan* was started. This plan aims to improve the mental health literacy by 70% for urban and 50% for rural residents by the end of 2020. Over the past 5 years, the government, professional institutes, and public media have expended tremendous effort to increase widespread publicity about mental health and psychological information in hospitals, schools, communities, enterprises, institutions, and different types of incarceration facilities (26). Thus, the higher level of depression literacy found in the present analysis might suggest a real increase in mental health literacy within the Chinese population, although more data and evidence are needed to support this speculation.

In contrast, bipolar disorder (including mania) has been reported to have the least correct identification for mental illnesses (3.3%). The correct rate of labeling bipolar disorder was as low as 5.7% in the survey of 212 Chinese participants recruited opportunistically from public places in Beijing (23). Our study suggested a relatively higher rate of correctly labeling the mania vignette (26.6%). The difference could be explained by the above-mentioned factors for labeling the depression vignette. However, compared with the 65.9% rate of correct labeling of the depression vignette, the participants had a much lower literacy for mania in this study. Only 36.4, 8.7, and 10.4% of participants had heard of mania, hypomania, and bipolar disorder respectively, which were far below the percentage for depression (86.1%). The complicated characteristics of bipolar disorder potentially contributed to the lower identification rate. Even for psychiatrists, bipolar disorder is frequently misdiagnosed or undiagnosed in China (19, 27, 28). A meta-analysis has reported that the estimated interval between the onset of bipolar disorder and its initial management was 5.8 years (29), and a survey from China reported the mean duration of undiagnosed bipolar disorder was 40.5 months (19). In our survey, mania is shown to be most frequently mislabeled as schizophrenia (39.9%). A possible explanation for the common mislabeling is that mania and schizophrenia share similar features, and the public in mainland China has more awareness of schizophrenia than bipolar disorder (23). This survey does not show any evidence of moderation effects resulting from gender or age on the recognition of depression and mania as reported in a recent nationwide study of mental health literacy in China (18).

The responses of participants to the helpfulness of different professionals and coping activities for the disorders depicted in the vignettes in this survey were compared with those in the previous relevant studies conducted in China (23). Professional help from a psychological therapist (or counselor) and a psychiatrist as well as social support from close families and friends were commonly recommended (41–42%). However, self-help by going out to travel was the most frequent recommendation (47.4%) by our participants, and 13.9% of respondents suggested “taking more rest.” Other self-help strategies, such as learning how to relax, doing physical exercise, and reading a self-help book, were also considered (30). Gong et al. found that seeking help from a psychologist/psychiatrist was the most highly recommended intervention for depression and the lowest for bipolar disorder (23). Thus, beliefs about the usefulness of professionals may be significantly different across illnesses. In addition, it may be very different across countries and cultures (23, 31). For example, in this study, 8.7% of respondents chose to seek help from a Chinese medical doctor for dealing with depression, whereas a previous study reported that more than 30% of Chinese people (Hong Kong 36.2%, Shanghai 39.5%, and Melbourne 31.8%, respectively), rated Chinese medical doctors as “helpful” (15).

The attitudes of participants in this study toward the usefulness of psychiatric treatment for mood disorders were very positive. Most respondents thought that long-term treatment of psychotropic medication and combination therapy of medication treatment and psychotherapy/psychological counseling would be necessary for patients diagnosed with mood disorders. However, in the real world, there are many factors affecting people's willingness to seek help or their intentions to use mental health services. An epidemiological survey in four provinces in China reported that the rate of seeking professional help is as low as 8% among individuals with a diagnosable mental disorder (2). The huge discrepancy between the potential need for psychiatric treatment and the actual behavior of seeking professional help could be attributed to the following barriers: the absence of knowledge about mental illness, negative attitudes about the mentally ill and psychotropic drugs, and inaccessibility of mental health services (23, 32–36).

In light of our findings and those of others, future research should aim to develop effective interventions for older people to increase their health literacy of mood disorder, reduce personal and perceived stigma, and enhance self-motivation and social support, which would improve attitudes, intentions, and help-seeking behaviors for mental health problems (37–39). Such programs may need to consider the cultural and socio-contextual factors (31). Social-cognitive intervention based on the theory of planned behavior as well as appropriate family support and services could be effective in the enhancement of mental health awareness and the promotion of mental health services (e.g., help-seeking behavior) in Chinese society (40–42). It is also necessary for current policies in China to pay more attention to enhancing the competency of primary-care health workers (e.g., general physicians) in order to provide adequate and convenient community-based mental health services to meet the needs of the increasing elderly populations (43, 44). Moreover, with the development of information and mobile technology, online education and intervention may facilitate help-seeking among individuals deterred by stigma (45).

There were several limitations in this study. First, the sample size was small, only those aged ≥60 years were enrolled, and the sample is limited to only two health centers in China, which limits the generalizability of the findings. Second, the participants were asked to choose responses from the given lists for most items, which means that the data did not capture anything else that participants may have thought. Third, the limited sample size was not enough to conduct more inferential statistical tests to compare the results between different groups, except stratifying by gender and age for the percentage of participants correctly labeling to the vignettes of depression and mania. Finally, our analyses cannot control for interactions between respondents and research psychiatrists, which may have potentially varied in the interpretation of the questions or answer options.

## Conclusion

In conclusion, this study demonstrates that the aged outpatients in community healthcare service settings in Shanghai have good depression literacy, including awareness of depression and correct identification of a depression vignette, but much more limited bipolar disorder (mania) literacy. Self-help strategies (e.g., going out to travel), seeking help from family and friends, and preferring to seek professional help from a psychologist (counselor) or a psychiatrist are the major strategies recommended by more than 40% of the participants. Moreover, the attitudes of most participants toward the usefulness of psychiatric treatment for mood disorders were very positive, and they thought that long-term treatment using psychotropic medication as well as combination therapy of medication treatment and psychotherapy/psychological counseling were both necessary for patients diagnosed with mood disorders.

## Data Availability Statement

All datasets generated for this study are included in the article/supplementary files.

## Ethics Statement

The study was approved by the Hongkou Mental Health Center Medicine Ethics Committee, Shanghai, China. The patients/participants provided their written informed consent to participate in this study.

## Author Contributions

LH and RH contributed to the participants' enrollment and the clinical assessments, and draft the manuscript. ZuW, ZhW, and YiF contributed to the design, analysis and interpretation of data, and revise the manuscript. YuF, WX, JX, and RM contributed to the participants' enrollment and the clinical assessments. TL, DM, and JC contributed to the interpretation of data, and revise the manuscript. All authors contributed to the article and approved the submitted version.

## Conflict of Interest

The authors declare that the research was conducted in the absence of any commercial or financial relationships that could be construed as a potential conflict of interest.
